# Factors associated with quality of life of adult patients with acute leukemia and their family caregivers in China: a cross-sectional study

**DOI:** 10.1186/s12955-020-1269-8

**Published:** 2020-01-07

**Authors:** Chunfeng Wang, Jie Yan, Jingyi Chen, Ying Wang, Ying Chun Lin, Rong Hu, Yong Wu

**Affiliations:** 1 School of Nursing, Fujian Medical University, No. 1 Xueyuan Road, Shangjie Town, Minhou County, Fuzhou City, 350108 Fujian Province China; 2 Department of Hematology and Rheumatology, the First Affiliated Hospital of Fujian Medical University, No. 20 of Chazhong Road, Taijiang District, Fuzhou City, 350004 Fujian Province China; 3 Department of Hematology, Fujian Medical University Union Hospital, No. 29 Xinquan Road, Gulou District, Fuzhou City, 350001 Fujian Province China

**Keywords:** Acute leukemia, Family caregiver, Quality of life, China

## Abstract

**Background:**

Acute leukemia (AL) not only impairs the quality of life (QOL) of patients, but also affects that of their family caregivers (FCs). Studies on QOL of AL patients and their FCs are limited. This study aimed to evaluate the QOL of AL patients and their FCs, and to explore the factors associated with QOL of patients and of FCs.

**Methods:**

A multicenter cross-sectional study was conducted. The QOL of 196 patient–FC dyads was assessed. The Functional Assessment of Cancer Therapy-Leukemia (FACT-Leu) was used for patients, and the 36-item Short-Form Health Survey (SF-36) was used for FCs. Independent-samples t-tests or one-way analysis of variance were used to compare QOL subscale scores between groups with different sociodemographic/clinical characteristics. Multiple regression analysis was conducted to identify the factors associated with QOL of AL patients and their FCs.

**Results:**

The total FACT-Leu score for AL patients was 76.80 ± 16.44, and the physical component summary (PCS) and mental component summary (MCS) scores for FCs were 64.67 ± 15.44 and 52.50 ± 13.49, respectively. All QOL subscales for patients (t = 12.96–34.73, *p* < 0.001) and FCs (t = 2.55–14.36, *p* < 0.05), except role emotional (t = − 0.01, *p* = 0.993), were lower than those reported in previous studies. Sex, employment, and chemotherapy were significantly associated with total FACT-Leu score in AL patients (*p* < 0.05). Age, sex, marital status, education, employment, and relationship to patients were significantly associated with SF-36 PCS or MCS (*p* < 0.05).

**Conclusions:**

AL patients and their FCs both have lower QOL than the population in previous studies. These findings suggest that not only AL patients’ physical and mental health but also overall family QOL should be assessed. Interventions supporting patient–FC dyads should be developed to improve their QOL.

## Background

Acute leukemia (AL) is characterized as a progressive malignant neoplasm of hematopoietic stem cells with acute onset, severe symptoms, poor survival rates, and frequent recurrence compared with solid tumors [1, 2]. According to Global Cancer Statistics 2018, leukemia is estimated to account for 437,033 new cases and 309,006 deaths in 185 countries, with a mortality rate of 3.2% [3]. Cancer statistics in China show approximately 75,300 new cases and 53,400 deaths of leukemia patients in 2015 [4]. Fatigue or lack of energy, pain, appetite loss, and insomnia affect 24–83% of patients with AL; this can significantly lower their health-related quality of life (QOL) [5]. In addition, the main treatment for AL is chemotherapy; this can cause a series of side effects including nausea and vomiting, anorexia, alopecia, and myelosuppression, which may further impair QOL [6]. The available evidence shows that almost all cancer patients, including those with blood cancers, experience a complex array of challenges that threaten their physical, psychological, and spiritual well-being [7–9].

In general, QOL assessment enables comprehensive evaluation of an illness’s interference with an individual’s adaptive functioning and can take into account the person’s values, perspectives, satisfaction, living conditions, accomplishments, functionality, cultural context, and spirituality. Specifically, health-related QOL is defined as the status of physical, emotional and psychological well-being [10]. It is recommended that health-related QOL is evaluated along with clinical and laboratory indicators [11]. Evaluating health-related QOL informs an integral part of treatment effectiveness [12] and burden of disease [13].

Families have an indispensable role in the treatment and rehabilitation of patients in China. Cancer is not only a personal experience of suffering, but also a family event. Influenced by Confucianism and the shortage of nursing professionals, family caregivers (FCs), usually the patient’s spouse or close relatives, play a critical part in taking care of patients, sometimes being forced to alter their own daily routines or even give up work as a result [14, 15]. Family members are often the major source of social and emotional support for Chinese leukemia patients [16]. Understandably, FCs in China are particularly exposed to the responsibility and burden of looking after their loved ones and witnessing what they experience during treatment. The negative experiences of FCs can not only impair their own QOL but may also compromise their ability to provide care [17, 18]. Importantly, the QOL of FCs caring for cancer patients in Asia is lower than that of their Western counterparts; this is mainly attributed to the ‘double torture’ of their natural duty, including filial piety and obligatory care, and the financial burden associated with national health insurance [19, 20]. Moreover, research has indicated that FCs caring for cancer patients often encounter serious physical and psychological issues, including anxiety, depression, helplessness, hopelessness, fatigue, fear, guilt, and sleep disorders, which further damage their QOL [21–25].

Although numerous studies have been conducted to identify factors associated with the QOL of cancer or chronic disease patients and their FCs, most of the cancer studies have involved mixed samples of patients with different types of cancer [26–31]. Studies have reported that patients’ QOL dimension scores (including physical health, physiological function, and mental health) are related to the QOL of FCs [32, 33]. Unlike solid tumors, AL follows an unpredictable trajectory, often involving acute deteriorations, so there are prognostic difficulties for AL patients [34]. Few studies have investigated the factors influencing the QOL of AL patients and that of their FCs.

In view of the limited literature on the QOL of AL patients and their FCs, this study aimed to evaluate the QOL of AL patient–FC dyads, identify the demographic and characteristic factors of contributing to the QOL of AL patients and their FCs.

## Methods

### Study design and participants

A multicenter cross-sectional survey was conducted at Fujian Medical University Union Hospital, the First Affiliated Hospital of Fujian University, and Fujian Provincial Hospital. The participating hospitals were selected because they are the three largest third-grade general hospitals in Fujian province.

Data were collected by four dedicated investigators through face-to-face interviews at each patient’s hospital bed from April 2017 to January 2018. When a patient and his/her FC were interested in participating, the patient–FC dyad was consulted and the patient’s and FC’s eligibility were assessed in accordance with the inclusion criteria. Importantly, only patient–FC dyads in which both the patient and the FC fulfilled the inclusion criteria were invited to participate. All participants provided written informed consent. The inclusion criteria for patients were as follows: (1) inpatients diagnosed with AL; (2) age ≥ 14 years; (3) had been informed of disease diagnosis and treatment; and (4) had complete ability to understand the questionnaire. The inclusion criteria for FCs were: (1) age ≥ 18 years; (2) a member of the patient’s family; (3) had primary responsibility for caring for the patient; and (4) had complete ability to understand the questionnaire.

The Functional Assessment of Cancer Therapy-Leukemia (FACT-Leu) was used to assess the AL patients’ QOL, and the 36-item Short-Form Health Survey (SF-36) was used to assess their FCs’ QOL. The participating AL patients and FCs were all encouraged to complete the questionnaire independently. However, assistance from the investigator was always available if necessary, for example, if the respondent had low visual acuity or another disability. After each participant handed in their questionnaire, the investigator reviewed it and asked the participant to complete any missing items.

Three hundred and six patient–FC dyads were approached, of which 92 dyads declined to participant owing to patients’ illness (*n* = 31), absence of FCs (*n* = 24), no interest in research (*n* = 18), no time (*n* = 13), or no reason (*n* = 6). Two hundred and fourteen patient–FC dyads completed their questionnaires, but 18 questionnaires contained missing data (regarding age, marital status, duration of caregiving, education, and relationship to patient). The 196 pairs of questionnaires without missing data were included in the final analyses. The effective questionnaire completion rate was 92.6%.

### Measurements

#### Participants’ sociodemographic and clinical characteristics

Self-designed questionnaires were used to collect data on the sociodemographic and clinical characteristics of patients and their primary FCs. The sociodemographic information on patients included age, sex, marital status, level of education, employment status, religious belief, and monthly household income per capita. The clinical characteristics included type of AL (acute lymphoblastic leukemia [ALL] or acute myelogenous leukemia [AML]), time since diagnosis, and phase of treatment (chemotherapy or non-chemotherapy treatment). The sociodemographic characteristics of primary FCs included age, sex, marital status, level of education, employment status, religious belief, relationship to the patient, and hours of caregiving per day.

#### Functional assessment of Cancer therapy-leukemia

The FACT-Leu questionnaire, which was designed to measure health-related QOL in leukemia patients, contains 27 Functional Assessment of Cancer Therapy – General items and 17 leukemia-specific items, comprising physical well-being, social/family well-being, emotional well-being, functional well-being, and leukemia-specific subscales. The internal consistency of the FACT-Leu total and subscale scores is high (Cronbach’s α ranges from 0.75 to 0.96). Test–retest reliability is adequate for all subscales (the intraclass correlation coefficient ranges from 0.77 to 0.89). The items are assessed according to a five-point Likert scale (0 = not at all; 1 = a little bit; 2 = somewhat; 3 = quite a bit; and 4 = very much), and item scores are summed to give the scores for each dimension and the total score; higher scores indicate better QOL. Importantly, the Chinese version of FACT-Leu has also been proved to be reliable for measuring the QOL of leukemia patients [35].

#### Medical outcomes study 36-item short-form health survey

The SF-36 questionnaire contains eight scales, summarized into two scores: physical component summary (PCS) and mental component summary (MCS) scores. PCS includes four scales: physical functioning, role physical, bodily pain, and general health perception. MCS includes four domains: vitality, social functioning, role emotional (RE), and mental health. Each scale consists of 2 to 10 items, each of which is evaluated on a 2–6-point Likert scale. Higher scores (ranging from 0 to 100) reflect better QOL. These summary scores give an overall assessment of QOL related to physical and mental health. Cronbach’s α of the Chinese PCS and MCS scales ranges from 0.85 to 0.87 [36]. Previous studies have shown that the SF-36 can be widely applied among people with chronic diseases and in healthy populations [37, 38], especially Chinese populations [39] and caregivers [40].

### Statistical analysis

Participants’ characteristics were described using frequencies or percentages for categorical variables, and means with standard deviations or medians with interquartile ranges (IQR) for continuous variables. QOL subscale scores were compared between groups with different sociodemographic and clinical characteristics using independent-samples t-tests (for two groups) or one-way analysis of variance (for multiple groups). The QOL of AL patients in this study was compared with that found by a previous study [41] conducted in leukemia patients using the FACT-Leu scale; the QOL of FCs was also compared with that of a previous study [42] in Chinese FCs caring for elderly people with chronic diseases using SF-36. Data from previous studies were all extracted from the published papers. The normality of the continuous variables was tested using the Kolmogorov–Smirnov test. Total and subscale scores of the FACT-Leu and SF-36 were calculated using scoring algorithms. Multiple linear regression analyses were conducted to assess predictors of patients’ total FACT-Leu score and FCs’ PCS and MCS scores. Sociodemographic and clinical variables for AL patients and FCs were also entered as independent variables. All tests were two-tailed, and a *p*-value of less than 0.05 was considered to be statistically significant. Data entry and statistical analyses were performed using SPSS version 22.0 for Windows (IBM Corp., Armonk, NY, USA).

## Results

### Participants’ characteristics

A total of 196 patient–FC dyads completed the survey. The mean age of the patients was 36.94 ± 14.00 years (ranging from 14 to 70 years), 54.6% were male, and 71.9% were married. Most patients were unemployed (89.8%) and unaffiliated with a religion (52.6%). The vast majority (83.7%) of the patients had a middle-school education level or higher. AML was more common (61.7%) than ALL (38.3%). The majority (81.1%) of patients were in the chemotherapy stage. About half (46.4%) of the patients were undergoing initial treatment. The median (IQR) duration since diagnosis was 5 (2–10) months. The median (IQR) monthly household income per capita was 3 (2–5) thousand Yuan, but only 92 patients completed this question. The median (IQR) medical cost was 150 (100–300) thousand Yuan.

The majority of FCs (53.6%) were female. The mean age of the FCs was 38.93 ± 10.69 years (ranging from 18 to 66 years). The majority of the FCs were spouses (46.9%) or parents (25%) of the patients and were married (87.8%), and 62.2% shared caregiving responsibilities with another person. Most were unemployed (67.9%) and affiliated with a religion (51%). Most FCs’ education levels were middle-school or higher (82.6%). The majority of FCs indicated that they completely (50.5%) or partially (46.9%) understood the patient’s disease. The median (IQR) number of hours of caregiving per day was 20 (10–24).

### QOL of AL patients and their FCs

Table 1 shows the QOL of AL patients and FCs. Compared with the results of a previous study [41], in which the FACT-Leu questionnaire was developed and validated among leukemia patients, the patients in this study had a significantly lower total QOL score and significantly lower scores in all domains (t = 12.96–34.91, *p* < 0.001). The FCs had significantly lower scores on all subscales excluding RE (t = –0.01, *p* = 0.993) compared with Chinese FCs caring for elderly people with chronic diseases (t = 2.55–14.36, *p* < 0.05) [42].

**Table 1:** Quality of life scores of patients and FCs (mean ± SD)

	Domain	Previous research [41, 42]	Current study	t	*p*
AL patients	PWB	22.9 ± 4.90	11.89 ± 6.31	24.42	< 0.001
(FACT-Leu)	SWB	24.7 ± 4.10	18.12 ± 4.68	19.66	< 0.001
	EWB	18.8 ± 4.20	8.63 ± 4.34	34.73	< 0.001
	FWB	18.3 ± 6.40	12.13 ± 6.66	12.96	< 0.001
	Leu subscale	52.1 ± 9.00	26.62 ± 10.22	34.91	< 0.001
	Total score	93.4 ± 18.90	76.80 ± 16.44	14.14	< 0.001
FCs	PF	90.65 ± 8.53	87.88 ± 15.22	2.55	0.012
(SF-36)	RP	52.64 ± 34.39	43.75 ± 43.44	2.87	0.005
	BP	84.60 ± 9.91	81.30 ± 17.97	2.57	0.011
	GH	52.36 ± 13.15	45.77 ± 9.04	10.21	< 0.001
	VT	66.52 ± 10.39	59.16 ± 14.31	7.20	< 0.001
	SF	75.71 ± 16.71	56.44 ± 19.30	13.98	< 0.001
	RE	40.96 ± 35.09	40.99 ± 41.25	−0.01	0.993
	MH	65.67 ± 11.86	53.45 ± 11.91	14.36	< 0.001
	PCS	70.06 ± 16.49	64.67 ± 15.44	4.88	< 0.001
	MCS	62.22 ± 18.51	52.5 ± 13.49	10.08	< 0.001

*PWB* physical well-being, *SWB* social/family well-being, *EWB* emotional well-being, *FWB* functional well-being, *PF* physical functioning, *RP* role physical, *BP* bodily pain, *GH* general health perception, *VT* vitality, *SF* social functioning, *RE* role emotional, *MH* mental health, *PCS* physical component summary, *MCS* mental component summary, *SD* standard deviation *AL* acute leukemia, *FC* family caregiver, *FACT-Leu* Functional Assessment of Cancer Therapy-Leukemia, *SF-36* 36-item Short-Form Health Survey

Table 2 shows the relationships between sociodemographic and clinical characteristics and QOL of patients and FCs. Total QOL score was significantly higher for female patients compared with male patients (t = − 2.03, *p* = 0.044). Employed patients had significantly lower QOL than those who were unemployed (t = − 3.234, *p* = 0.001). There was a significant difference in QOL by phase of treatment (chemotherapy or non-chemotherapy treatment) (t = − 2.03, p = 0.044). However, there was no difference in the QOL of patients during initial and non-initial treatment (t = 0.680, *p* = 0.498). There was also no difference in QOL among patients with FCs who had different relationships with the patients (F = 1.126, *p* = 0.346). For FCs, the PCS score was significantly higher for FCs aged < 45 years than for those of other ages (F = 3.798, *p* = 0.024). Married FCs had significantly lower PCS (t = 3.758, *p* < 0.001) and MCS (t = 4.626, p < 0.001) scores than unmarried FCs. The PCS score significantly increased as the educational level of the FCs increased (F = 9.299, p < 0.001). Employed FCs had a significantly higher PCS score than unemployed FCs (t = 2.722, *p* = 0.007). Parents had the lowest PCS (F = 4.644, *p* = 0.001) and MCS (F = 4.621, p = 0.001) scores compared with FCs with other relationships to patients. Moreover, female FCs had a significantly higher MCS score (t = − 3.573, *p* = 0.004).

**Table 2:** Relationships between sociodemographic and clinical characteristics and quality of life of patients and FCs

Characteristic	Total FACT-Leu score (Patients)			SF-36 PCS score (FCs)			SF-36 MCS score (FCs)		
	Mean ± SD	Statistic	*p*	Mean ± SD	Statistic	*p*	Mean ± SD	Statistic	*p*
Sex		−2.03 (t)	0.044		−0.004 (t)	0.997		−3.573 (t)	0.004
Male	74.65 ± 16.41			64.67 ± 14.76			48.92 ± 13.86		
Female	79.39 ± 16.19			64.68 ± 16.09			55.62 ± 12.40		
Age (years)		2.259 (F)	0.107		3.798 (F)	0.024		2.587 (F)	0.078
< 45	75.38 ± 16.11			66.88 ± 15.49			54.16 ± 14.33		
45–59	81.39 ± 16.20			60.52 ± 14.83			49.59 ± 11.54		
60–74	76.33 ± 18.70			65.75 ± 13.36			50.72 ± 11.33		
Marital status		−0.793 (t)	0.429		3.758 (t)	< 0.001		4.626 (t)	< 0.001
Married	77.38 ± 16.31			63.18 ± 15.42			50.93 ± 12.94		
Unmarried	75.31 ± 16.81			75.42 ± 10.80			63.86 ± 11.98		
Educational level		0.132 (F)	0.876		9.299 (F)	0.000		1.578 (F)	0.209
Primary school or below	75.75 ± 12.86			55.96 ± 14.81			51.25 ± 12.47		
Middle or high school	77.29 ± 18.21			64.85 ± 14.85			51.57 ± 13.16		
University or higher	76.37 ± 14.38			70.10 ± 14.78			55.38 ± 14.64		
Religious belief		0.039 (t)	0.969		0.332 (t)	0.740		−0.463 (t)	0.644
Yes	76.75 ± 16.12			64.32 ± 17.16			52.95 ± 13.48		
No	76.85 ± 16.79			65.05 ± 13.51			52.05 ± 13.55		
Employment		−3.234 (t)	0.001		2.722 (t)	0.007		0.893 (t)	0.373
Employed	65.80 ± 16.66			68.97 ± 14.66			53.76 ± 14.70		
Unemployed	78.05 ± 15.99			64.64 ± 15.44			51.92 ± 12.89		
Type of leukemia		−0.948 (t)	0.344						
AML	77.68 ± 17.14								
ALL	75.39 ± 15.24								
Treatment		−2.030 (t)	0.044						
Chemotherapy	75.66 ± 15.62								
Non-chemotherapy	81.70 ± 19.03								
Undergoing initial treatment		0.680 (t)	0.498						
Yes	77.66 ± 15.43								
No	76.06 ± 17.30								
Relationship to patient		1.126 (F)	0.346		4.644 (F)	0.001		4.621 (F)	0.001
Spouse	78.17 ± 13.88			63.33 ± 15.13			51.66 ± 13.04		
Parent	73.06 ± 18.39			60.61 ± 15.67			49.26 ± 13.61		
Child	79.02 ± 19.30			67.99 ± 14.37			53.87 ± 13.75		
Sibling	72.14 ± 20.68			74.29 ± 15.78			62.78 ± 8.25		
Other	76.57 ± 6.58			81.79 ± 2.27			68.23 ± 1.66		
Understanding of the disease					0.726 (F)	0.458		1.640 (F)	0.197
Incomplete				59.50 ± 16.29			42.49 ± 7.42		
Partial				63.73 ± 16.11			52.11 ± 14.77		
Complete				65.82 ± 14.80			53.39 ± 12.29		

*ALL* acute lymphoblastic leukemia, *AML* acute myelogenous leukemia, *SD* standard deviation, *PCS* physical component summary, *MCS* mental component summary, *FC* family caregiver, *FACT-Leu* Functional Assessment of Cancer Therapy-Leukemia, *SF-36* 36-item Short-Form Health Survey

### Factors associated with QOL of AL patients and FCs

Total FACT-Leu score and SF-36 PCS and MCS scores were used as dependent variables. Multiple linear regression analyses were conducted to assess the relationships between potentially associated factors and dependent variables. First, AL patients’ age, educational level, employment, and average monthly household income per capita were entered into the model to determine which factors explained the variance in the QOL of AL patients. Of these four variables, age and employment explained the largest proportion of the variance (total FACT-Leu score model: F = 2.759, *p* = 0.033, R^2^ = 0.113, adjusted R^2^ = 0.072). The results are shown in Table 3. Second, FCs’ age, educational level, type of caregiving (independent or assisted by others), understanding of the disease, relationship to patient, and MCS score were entered into the PCS model. Of these variables, education and MCS score explained the largest proportion of the variance (PCS model: F = 30.703, *P* = 0.000, R^2^ = 0.494, adjusted R^2^ = 0.478). Third, FCs’ age, sex, educational level, type of caregiving (independent or assisted by others), understanding of the disease, relationship to patient, and PCS score were entered into the MCS model. Of these variables, FCs’ sex and PCS score explained the largest proportions of the variance (MCS model: F = 23.626, *p* < 0.001, R^2^ = 0.468, adjusted R^2^ = 0.448). The results are shown in Table 4.

**Table 3:** Factors associated with quality of life of AL patients

Predictors	Total FACT-Leu score		
	Adjusted β	t-value	*p*
Age	0.214	1.993	0.049
Educational level	0.097	0.896	0.373
Employment	0.203	2.002	0.048
Average monthly household income per capita	0.192	1.852	0.067
R^2^	0.113		
Adjusted R^2^	0.072		

*AL* acute leukemia, *FACT-Leu* Functional Assessment of Cancer Therapy-Leukemia

**Table 4:** Factors associated with quality of life of FCs

Predictors	SF-36 PCS			SF-36 MCS		
	Adjusted β	t-value	*p*	Adjusted β	t-value	*p*
Age	−0.028	−0.513	0.608	0.008	0.143	0.887
Sex				0.241	4.177	< 0.001
Educational level	0.204	3.823	< 0.001	−0.042	−0.729	0.467
Understanding of disease	0.024	0.452	0.652	0.038	0.705	0.482
Relationship to patient	0.068	1.223	0.223	0.011	0.195	0.846
Type of caregiving	−0.009	−0.166	0.868	−0.022	− 0.400	0.690
PCS				0.643	11.216	< 0.001
MCS	0.625	11.521	< 0.001			
R^2^	0.494			0.468		
Adjusted R^2^	0.478			0.448		

*FC* family caregiver, *SF-36* 36-item Short-Form Health Survey, *PCS* physical component summary, *MCS* mental component summary

## Discussion

### Participants’ QOL

To the best of our knowledge, this study is the first attempt in mainland China to concurrently examine the QOL of both patients with AL and their FCs. The findings of this study indicate that AL patients have a lower QOL on all subscales compared with those reported by a previous study conducted in the USA [41]. The lower scores across all the QOL domains may have been due to differences in particular sociodemographic and clinical characteristics (including treatment and the predictability of cancer progression) of the samples in the two studies. In particular, most patients in the present study were males (54.6%) aged < 45 years (69.9%), who tend to have lower QOL than other demographic subgroups. This is consistent with a study in Iran, which showed that patients with AL had poor QOL, especially male patients [43], possibly owing to traditional cultural practices in which males have more family responsibilities and other obligations. The employed patients (10.2%) had significantly lower QOL compared with the unemployed patients, possibly because working aggravated the patients’ fatigue and physical burden. Patients undergoing chemotherapy had lower QOL, because adverse reactions to chemotherapy aggravate physical burdens [44]. Surprisingly, there was no difference in QOL between patients undergoing initial and non-initial treatment, contrary to the results of a study conducted in the USA [6]. Although the differences were not significant, patients cared for by parents and siblings had lower QOL than other patients, suggesting that close FC relationships may influence patients’ QOL. A larger study is needed to verify this result.

The FCs had lower QOL (in each domain except RE) compared with Chinese FCs caring for elderly people with chronic diseases [42]. These findings may be attributed to the following factors. First, AL symptoms are more serious and complex than those of many chronic diseases. Second, about half (46.9%) of the FCs of the AL patients were spouses, whereas the majority of FCs caring for the elderly are adult children [42]. Similar research indicated that FCs who were the spouses of the patients they were caring for had lower QOL than other FCs [45]. In our study, married FCs and FCs aged ≥45 years had worse QOL. This is consistent with the findings of another study of FCs caring for cancer patients, which showed that younger FCs had better QOL [46]. Increased educational level also led to better QOL, suggesting that FCs with more education may have more access to health-related information and pay more heed to their physical and mental health. On the other hand, those with less education have limited access to health-related information and may have insufficient incomes to deal with the changes brought about by disease. This reminds us that particular attention should be paid to those from lower-income families and those with lower educational levels; this may include providing them with the necessary information and emotional and financial support. Significantly, employed FCs had better PCS than those who were unemployed. This may be partly explained by the fact that employed FCs have fewer caring responsibilities, leaving them with more time for their usual activities and for relaxation. In addition, as parents had the lowest PCS and MCS scores compared with FCs with other relationships to the patients, the parents of patients need more supportive care.

### Factors associated with patients’ and FCs’ QOL

The factors associated with total FACT-Leu score included patients’ age and employment. This revealed that the QOL of AL patients increased with age. However, this was inconsistent with the results of the univariate analysis. This may be due to the choice of age subgroups and the limited sample size. Meanwhile, this finding also highlights that it is important for patients to rest instead of continuing to work after the diagnosis of disease. Unexpectedly, only 92 patients completed the item on household income. This may have been partly owing to the conservative culture in China that leads to a reluctance to disclose one’s financial status, as well as the inconvenience of calculating one’s household income. This suggests that the average monthly household income per capita is not the best indicator by which to evaluate the family economic situation of patients. The average monthly household income per capita in this study was 3000 Yuan, which is below the average income in China. This may be partly explained by the fact that the majority of patients and their FCs were unemployed and thus did not have a steady income to pay for expensive treatments. The results indicated that financial difficulties related to the burden of AL were likely to impair patients’ QOL, consistent with other studies that found financial difficulties to be a strong predictor of impaired health status among cancer patients [47, 48].

Notably, our study indicated that FCs’ educational level and MCS score were significantly associated with their PCS score. A higher educational level led to higher QOL among FCs, possibly owing to stronger self-adjustment and adaptability. Generally, people with higher education earn relatively higher incomes and have fewer financial burdens. A poorer PCS was associated with a poorer MCS owing to the burden of symptoms and psychological stress, which indicated that the two scores could be used to predict each other. This highlights the importance of FCs’ mental well-being as well as their physical well-being. Moreover, PCS score was also associated with FCs’ sex and marital status. Female and unmarried FCs had higher QOL. The traditional Chinese view is that unmarried and female individuals have fewer family responsibilities and burdens, which may explain their higher QOL. Particular attention should be paid to married and male FCs, especially those with poor physical health.

### Limitations

The present study had several limitations. First, the survey was conducted in only one province, which limits the generalizability of the findings. However, the findings regarding the factors associated with the QOL of FCs are unlikely to be seriously influenced by sample selection bias. Second, the sample size was relatively small, because it is challenging to recruit patient–FC dyads given that not one but two individuals must independently agree to participate. In addition, as different instruments were used to assess the QOL of patients and FCs, no direct comparison between them could be made.

## Conclusions

Our findings have clear clinical implications regarding the focus of interventions. First, AL patients and their FCs had lower QOL than other patient groups; this was considered to be because the trajectory of AL is unpredictable and AL patients often experience acute deteriorations and serious symptom burdens. This clearly indicates that intervention for AL patients and their FCs is essential. Second, FCs’ general health should be considered during the course of treatment and nursing. Not only patients’ physical and mental health but also overall family well-being should be assessed. Cancer is the most researched area in artificial intelligence (AI) in Medicine [49]. Tailored interventions such as using AI to provide information and emotional support to patient-FC dyads should be developed to improve the QOL of this group. Finally, future studies should explore more factors that potentially underlie the QOL of patient–FC dyads, such as family function, therapeutic regimen, financial difficulties, and comorbidities.

## Data Availability

The datasets generated during and/or analyzed during the current study are available from the corresponding authors on reasonable request.

## Abbreviations

AL: Acute leukemia
ALL: Acute lymphoblastic leukemia
AML: Acute myelogenous leukemia
FACT-Leu: Functional Assessment of Cancer Therapy-Leukemia
FC: Family caregiver
IQR: Interquartile range
MCS: Mental component summary
PCS: Physical component summary
QOL: Quality of life
RE: Role emotional
SF-36: 36-item Short-Form Health Survey
